# Psychosocial and Occupational Factors Associated with Multisite Musculoskeletal Pain Among Motorcycle Couriers: A Cross-Sectional Study

**DOI:** 10.3390/healthcare14213814

**Published:** 2026-11-09

**Authors:** Adem Çalı, Tuana Saygı, Ömer Kaan Özmen, Oğuz Sinan Saatci, Javidan Abdullazade, Ömer Şevgin

**Affiliations:** 1 Department of Physiotherapy and Rehabilitation, Faculty of Health Sciences, İstanbul Topkapı University, 34020 Istanbul, Türkiye; 2 Department of Physiotherapy and Rehabilitation, Faculty of Health Sciences, Üsküdar University, 34662 Istanbul, Türkiye; tuanasaygii00@gmail.com (T.S.); omerkaan_ozmen@hotmail.com (Ö.K.Ö.); sinan.saatci.2004@gmail.com (O.S.S.); javidan.abdullazade@hotmail.com (J.A.); omer.sevgin@uskudar.edu.tr (Ö.Ş.)

**Keywords:** motorcycle couriers, musculoskeletal pain, Nordic Musculoskeletal Questionnaire, burnout, personal accomplishment, perceived stress, occupational health

## Abstract

**Background:** Motorcycle couriers face combined biomechanical and psychosocial demands, but the relationship between multisite musculoskeletal pain, perceived stress, and occupational burnout in this workforce remains insufficiently characterized. We aimed to determine the distribution of musculoskeletal pain and identify factors associated with multisite pain in this group. **Methods:** This cross-sectional study included 100 male motorcycle couriers working in Istanbul, Türkiye. Musculoskeletal symptoms in nine anatomical regions were assessed with the Nordic Musculoskeletal Questionnaire (12-month reference period); multisite pain was defined as pain in ≥3 regions. Perceived stress (14-item Perceived Stress Scale, PSS-14; one-month reference period) and the three Maslach Burnout Inventory dimensions were measured. Analyses comprised group comparisons, Spearman correlations, univariate and adjusted logistic regression—in which the non-linearity of the stress–pain association was modelled explicitly with a quadratic term—and sensitivity analyses (alternative thresholds, count models, education adjustment, and bootstrap validation). **Results:** Thirty-two percent reported pain in ≥3 regions; the neck (38%) and lower back (35%) were most affected. PSS-14 scores were markedly higher in the multisite pain group (median 45.0 vs. 26.0, *p* < 0.001) and correlated with the number of painful regions (ρ = 0.47). The stress–pain association was non-linear: the prevalence of multisite pain was 11.5%, 11.1%, 25.9%, and 95.0% across ascending PSS-14 quartiles, and the adjusted odds rose steeply only at high stress levels (highest quartile, PSS-14 ≥ 43). In the primary adjusted model including linear and quadratic PSS-14 terms, perceived stress remained independently associated with multisite pain (quadratic term *p* = 0.005; optimism-corrected AUC = 0.851; Hosmer–Lemeshow *p* = 0.43), and the association persisted after additional adjustment for educational level. Reduced Personal Accomplishment and Depersonalization were associated with the outcome only in unadjusted analyses; Emotional Exhaustion showed no detectable association. **Conclusions:** Multisite musculoskeletal pain affected one-third of motorcycle couriers and was strongly associated with perceived stress, in a threshold-like, non-linear pattern concentrated at high stress levels. Given the cross-sectional design and self-reported measures, longitudinal confirmation is needed; nevertheless, the findings support combining ergonomic measures with strategies addressing psychosocial strain in this workforce.

## 1. Introduction

Platform-mediated gig work has expanded rapidly worldwide, and app-based food and package delivery has become one of its most visible segments [1]. Recent systematic reviews of last-mile delivery workers indicate that this workforce operates under economic insecurity, algorithmic management, and sustained time pressure—conditions consistently linked to stress, fatigue, and adverse safety outcomes [2,3]. Motorcycle couriers represent a distinctive occupational group within this sector because their work combines prolonged sitting, constrained riding postures, frequent upper-extremity activity, exposure to whole-body vibration, repeated mounting and dismounting, traffic-related demands, and pressure to complete deliveries within limited time windows [3,4].

The musculoskeletal burden of this workforce is increasingly well documented. A recent systematic review and meta-analysis of 23 studies including more than 150,000 gig-based food-delivery workers reported pooled prevalences of 43% for lower-back, 39% for shoulder, 30% for neck, and 24% for upper-back complaints [5]. Consistent findings have been reported at the national level: work-related musculoskeletal disorders affected 62.1% of motorcycle food-delivery riders in Thailand [4], 74.9% of riders in eastern Malaysia [6], and 54.9% of takeaway riders in Shanghai [7], while approximately half of app-based riders in southern India reported lower-back pain [8]. Among platform riders in Milan, 59% reported back pain and almost half reported fatigue [9]. A recent study of occupational two-wheeler delivery drivers in Mumbai found a high burden of musculoskeletal disorders and demonstrated an association between psychological distress and the number of affected body regions [10]. Studies of motorcyclists more broadly have likewise identified the neck and lower back as particularly vulnerable regions [11], and motorbike drivers demonstrate greater neck pain and reduced cervical mobility than car drivers [12].

These symptom patterns are biomechanically plausible. Prolonged and static sitting postures are associated with low-back pain in occupational settings [13], and occupational whole-body vibration has been associated with low-back pain in a recent systematic review [14]. A meta-analysis of professional drivers reported a 12-month low-back-pain prevalence of 53%, with uncomfortable seats, improper driving posture, long working hours, and high work stress among the identified risk factors [15]. In motorcycle riders specifically, ergonomic modeling indicates that handlebar and seating geometry substantially determine postural discomfort [16], and whole-body vibration exceeding regulatory action values approximately tripled the odds of musculoskeletal disorders among motorcycle delivery riders [6].

The Nordic Musculoskeletal Questionnaire (NMQ) is widely used to identify musculoskeletal symptoms in nine major anatomical regions and is particularly suitable for epidemiological and occupational-health research. The original instrument was developed by Kuorinka et al. and has demonstrated acceptable reliability for identifying musculoskeletal symptoms in occupational settings [17]. The Turkish version has demonstrated appropriate test–retest reliability, internal consistency, and construct validity [18], and recent psychometric evaluations continue to support the reliability and validity of the Nordic framework in contemporary occupational samples [19].

Occupational musculoskeletal symptoms frequently involve more than one anatomical region, and multisite pain appears to constitute a distinct entity rather than the simple sum of single-site problems: in the multinational CUPID study, pain affecting many anatomical sites showed different risk-factor associations—particularly stronger links with psychosocial characteristics—than pain restricted to one or a few sites [20]. Multisite musculoskeletal pain is common even in the general population, with a 12-month prevalence of 46.5% reported in a recent population-based survey [21], and it predicts unfavorable outcomes, including sickness absence among younger employees [22] and accelerated disability trajectories [23]. Longitudinal occupational studies have shown that mental stress, hurry, poor workplace relationships, repetitive work, and awkward postures predict the development and persistence of multiple-site pain [24,25], and that psychosocial work factors prospectively influence the number of painful body sites [26].

**Figure 1:**
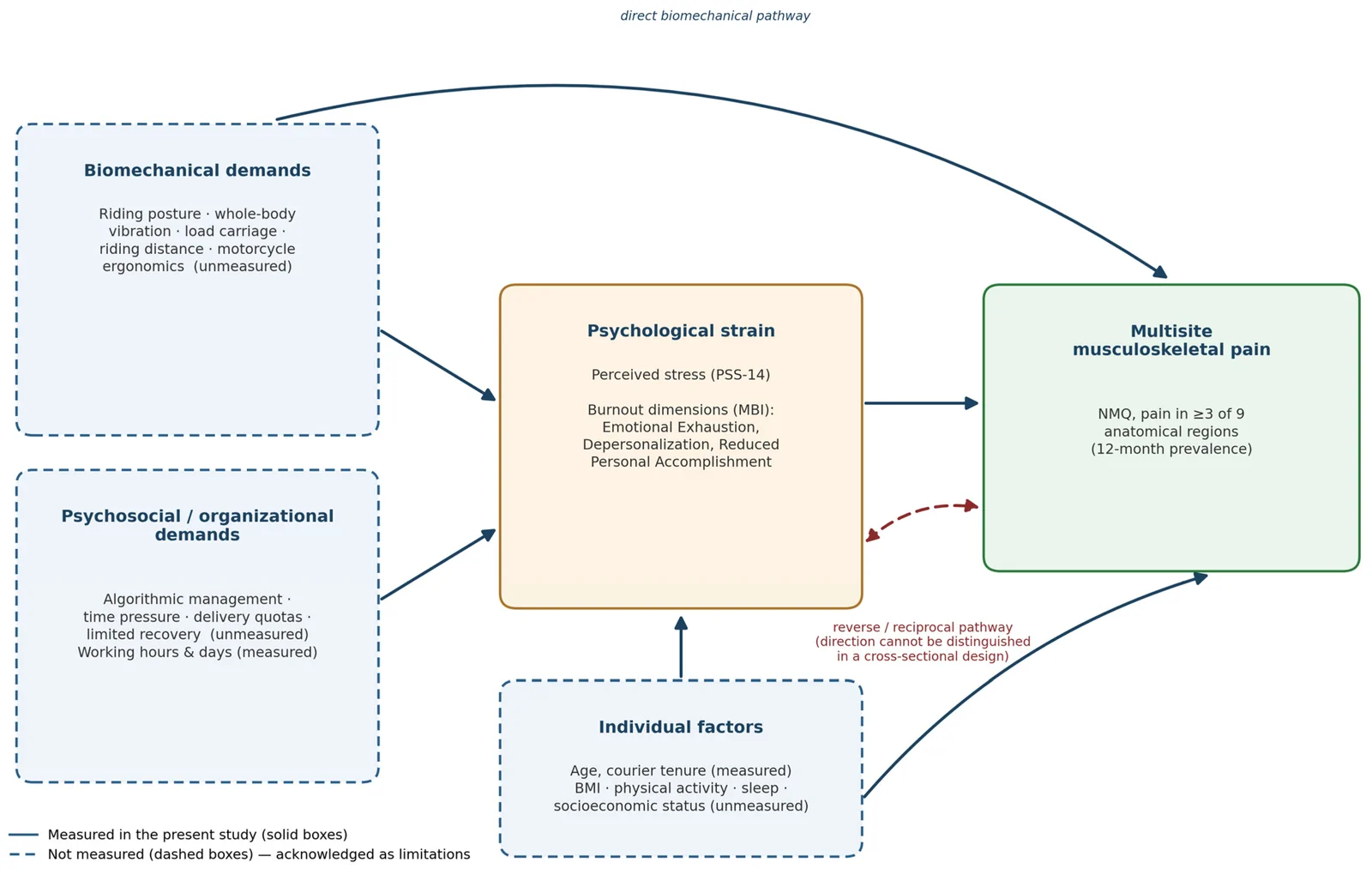
Conceptual framework of the hypothesized relationships between occupational demands, psychological strain, individual factors, and multisite musculoskeletal pain in motorcycle couriers, informed by the Job Demands–Resources model [29] within a biopsychosocial perspective. Solid boxes denote constructs measured in the present study; dashed boxes denote constructs that were not measured and are considered in the Section Limitations. The dashed red arrow denotes the reverse/reciprocal pathway, which cannot be distinguished from the forward pathway in a cross-sectional design. The framework is heuristic: the depicted pathways guided variable selection and interpretation but were not formally tested in this study. Solid arrows denote the hypothesized forward pathways from occupational demands and individual factors to psychological strain and multisite pain.

Accordingly, contemporary occupational-health models recognize that musculoskeletal symptoms cannot be understood exclusively through biomechanical exposure. A systematic review of longitudinal studies concluded that high job demands, low job control, and low support are associated with the development of musculoskeletal disorders [27], and a recent meta-analysis suggests that work-related psychosocial hazards contribute to musculoskeletal risk to a degree comparable with biomechanical hazards [28]. These strands can be integrated within the Job Demands–Resources (JD-R) model, in which sustained job demands—biomechanical as well as psychosocial—initiate a health-impairment process through strain, while inadequate job resources amplify it [29]. Applied to platform-based courier work from a biopsychosocial perspective, biomechanical demands are expected to act on musculoskeletal symptoms both directly and through psychological strain, with perceived stress and the burnout dimensions serving as strain indicators and multisite pain as the health-impairment outcome; the hypothesized relationships among the measured and unmeasured constructs of the present study, structured according to this framework, are shown in Figure 1. Burnout is a multidimensional occupational phenomenon encompassing emotional exhaustion, depersonalization or cynicism, and reduced personal accomplishment or professional efficacy; these dimensions should be considered separately rather than combined into a single total burnout score [30,31]. The personal-accomplishment dimension is conceptually and empirically distinct, showing the strongest inverse association with occupational self-efficacy among the three dimensions in meta-analysis [32]. Perceived stress has likewise been implicated in the development of chronic musculoskeletal pain [33], and psychosocial stress and musculoskeletal pain cluster in physically demanding occupations [34].

Motorcycle couriers may be particularly exposed to such psychosocial demands because platform work involves algorithmic performance monitoring, ranking and penalty systems, unpredictable traffic conditions, customer interactions, and limited recovery periods [2,3,35]. In a survey of 953 food-delivery riders, almost three-quarters experienced at least moderate burnout across all three Maslach dimensions, driven largely by algorithmic management features [35]. Algorithmic control has been linked to impaired mental health and risky riding through increased work pressure [36], labor intensity adversely affects riders’ safety and health through job pressure [37], and stress-related factors and fatigue predict occupational crashes in this workforce [38]. Nevertheless, evidence examining burnout dimensions together with multisite musculoskeletal pain in motorcycle couriers remains scarce; the study of Mumbai two-wheeler delivery drivers, which linked psychological distress to the number of affected body regions, remains among the few direct antecedents [10].

Therefore, the primary aim of the present study was to determine the distribution of musculoskeletal pain among motorcycle couriers and to investigate factors associated with multisite musculoskeletal pain, operationally defined as pain affecting three or more NMQ anatomical regions. A secondary aim was to evaluate whether perceived stress and the individual dimensions of occupational burnout were associated with a greater musculoskeletal pain burden. We hypothesized that greater perceived stress and greater burnout-related impairment would be associated with increased odds of multisite musculoskeletal pain. These hypotheses follow directly from the framework in Figure 1: perceived stress and the burnout dimensions were conceptualized as indicators of the strain pathway linking occupational demands to musculoskeletal outcomes [29], perceived stress—the most proximal global strain appraisal—was expected to show the strongest association, and multisite pain was selected as the outcome because psychosocial factors show stronger associations with multisite than with localized pain [20].

## 2. Materials and Methods

### 2.1. Study Design and Ethics

This cross-sectional observational study was conducted among motorcycle couriers working in Istanbul, Türkiye, between January 2026 and June 2026. The study was approved by the Non-Interventional Research Ethics Committee of Üsküdar University (meeting no. 12, 26 November 2025; decision no. 61351342/020-1610) and was conducted in accordance with the Declaration of Helsinki. Written informed consent was obtained from all participants before enrolment and before any study-specific assessment was performed. The study is reported in accordance with the STROBE (Strengthening the Reporting of Observational Studies in Epidemiology) recommendations for cross-sectional studies.

### 2.2. Participants and Sampling

A total of 117 male motorcycle couriers actively working in Istanbul were approached and interviewed. Participants were recruited using a combination of convenience and snowball (chain-referral) sampling: couriers were approached in person at delivery waiting points and workplaces across the city, and participating couriers additionally referred other potentially eligible motorcycle couriers from their professional networks; this process was continued until the target sample size was reached. Inclusion criteria were active employment as a motorcycle courier for at least one year, age ≥ 18 years, use of a motorcycle as the primary vehicle for occupational delivery, and voluntary participation. Of the 117 couriers approached, 9 declined to participate and 8 were excluded because they had less than one year of experience as a motorcycle courier, leaving 100 participants (all male) in the final analysis. All 100 participants had complete data for the variables included in the present analysis. No formal a priori sample-size calculation was performed. The target of 100 participants was specified before data collection on combined feasibility and analytic grounds: it reflected the number of eligible couriers realistically reachable through the convenience and chain-referral process within the study window, and it was expected to support an adjusted logistic model with approximately three covariates under the events-per-variable principle, assuming an outcome prevalence of roughly one-third. The 32 outcome events ultimately observed confirmed that model complexity had to remain deliberately restricted (Section 2.7), and the resulting limits on precision are acknowledged explicitly in the Section Limitations.

### 2.3. Sociodemographic and Occupational Characteristics

A structured participant information form was used to record age, marital status, educational level, duration of employment as a motorcycle courier, daily working-hours category, and number of working days per week. Age and duration of courier employment were treated as continuous variables. Educational level was recorded as the highest level of completed education (middle school, high school, or university), and marital status (single/married), daily working hours (8–10 h or >10 h per day), and weekly working days (5, 6, or 7 days) were recorded as categorical variables. Marital status was used solely to describe the sample. Educational level was likewise summarized descriptively and was not included in the primary model because of unevenly distributed categories; however, given the observed between-group imbalance, it was examined in dedicated sensitivity analyses—primarily with conventional indicator (dummy) coding using middle school as the reference category, and additionally with the originally planned binary contrast (high school vs. middle school or university), which had been specified to preserve cell sizes given the small university category (n = 11) (Section 2.7).

### 2.4. Nordic Musculoskeletal Questionnaire

Musculoskeletal symptoms were evaluated using the NMQ, which assesses symptoms in nine anatomical regions: neck, shoulders, upper back, elbows, wrists/hands, lower back, hips/thighs, knees, and ankles/feet. The NMQ was originally developed for the standardized assessment of musculoskeletal symptoms in epidemiological and occupational settings [17], and the Turkish version has demonstrated satisfactory psychometric properties [18]. In the present study, only the general 12-month prevalence item of the questionnaire was analyzed: for each region, participants indicated whether they had experienced ache, pain, or discomfort during the previous 12 months, and responses were recorded dichotomously (1 = symptoms present; 2 = symptoms absent). The 7-day prevalence and activity-limitation items, and information on symptom severity or duration, were not included in the present analysis. For each participant, the number of anatomical regions with reported pain was summed, producing an NMQ painful-region count ranging from 0 to 9. For the primary analysis, participants were classified into two groups: 0–2 painful regions (lower musculoskeletal pain burden) and ≥3 painful regions (multisite musculoskeletal pain), a threshold consistent with prior studies of multisite pain in occupational populations [20,26]. Multisite musculoskeletal pain constituted the event category in the logistic regression analyses.

### 2.5. Perceived Stress Scale

Perceived stress was measured using the 14-item Perceived Stress Scale (PSS-14) developed by Cohen et al. [39]. The instrument measures the extent to which individuals perceive situations in their lives as unpredictable, uncontrollable, or stressful, with items answered in reference to the respondent’s thoughts and feelings during the last month. Items are scored on a five-point response scale (0–4), and positively worded items are reverse scored. Total scores range from 0 to 56, with higher scores representing greater perceived stress. The Turkish adaptation of the PSS-14 was conducted by Eskin et al. and demonstrated satisfactory reliability and validity, with a reported internal consistency coefficient of 0.84 [40]. The total PSS-14 score was treated as a continuous variable in all analyses. Because the PSS-14 refers to the last month whereas the NMQ refers to the previous 12 months, the temporal correspondence between the exposure and outcome measures is imperfect; the implications of this mismatch are considered in the Section Limitations.

### 2.6. Maslach Burnout Inventory

Occupational burnout was evaluated using the 22-item Maslach Burnout Inventory (MBI) [30,31] in its Turkish adaptation by Ergin, in which items are scored on a five-point scale (0–4) [41]. Burnout was analyzed according to its three component dimensions: Emotional Exhaustion (nine items; range 0–36), Depersonalization (five items; range 0–20), and Personal Accomplishment (eight items; range 0–32). The MBI conceptualizes burnout as a multidimensional construct, and the three dimensions are recommended to be considered separately rather than combined into a single global burnout score [30,31]; accordingly, no combined MBI total score was used in inferential analyses. In the present study, the Personal Accomplishment dimension was reverse-oriented and analyzed as Reduced Personal Accomplishment, such that higher scores represented a greater sense of occupational inefficacy and therefore a less favorable burnout-related profile. Consequently, higher Emotional Exhaustion represents greater burnout-related exhaustion, higher Depersonalization represents greater burnout-related detachment/cynicism, and higher Reduced Personal Accomplishment represents greater burnout-related inefficacy.

### 2.7. Statistical Analysis

Data quality was evaluated before inferential analyses: no missing data were identified, NMQ responses contained only the predefined binary codes, MBI subscale totals were mathematically verified, and all scale scores were re-checked against the original paper forms before the final analysis. The primary outcome (multisite musculoskeletal pain) and the primary exposures (PSS-14 and the three MBI dimensions) were specified a priori in accordance with the study hypothesis; the correlation analyses were considered exploratory, no formal adjustment for multiple comparisons was applied, and secondary findings should be interpreted accordingly. Continuous variables were examined for distributional characteristics using the Shapiro–Wilk test. Because several variables demonstrated departures from normality, continuous variables were summarized using the median and interquartile range (IQR) and compared between the two NMQ pain groups using the Mann–Whitney U test, with the rank-biserial correlation reported as the effect size. Categorical variables were reported as frequencies and percentages and compared using Pearson’s chi-square test. Associations between the psychosocial scale scores and the number of painful anatomical regions were examined using Spearman rank correlations.

Binary logistic regression was used to identify factors associated with pain in ≥3 anatomical regions. Separate univariate logistic regression models were fitted for age, years working as a courier, PSS-14 score, Emotional Exhaustion, Depersonalization, Reduced Personal Accomplishment, daily working-hours category, and weekly working days. Crude odds ratios (ORs), 95% confidence intervals (CIs), and *p*-values were calculated. In the adjusted analyses, Reduced Personal Accomplishment was entered together with age and the PSS-14 score as a priori covariates; model complexity was deliberately restricted because only 32 participants experienced the primary outcome. The linearity of the association between PSS-14 and the outcome on the logit scale was examined before model finalization, by modeling PSS-14 quartiles and by adding a quadratic term to the continuous specification. Because these checks demonstrated a clearly non-linear association, the primary adjusted model included both a linear and a quadratic term for PSS-14, with the score mean-centred before squaring to reduce collinearity; a quartile-based categorical specification and the linear-only model are reported alongside it, the latter as a comparison whose single odds ratio represents an average effect that does not apply uniformly across the score range. Age and duration of courier employment were not entered simultaneously because of their strong correlation (Spearman ρ = 0.86) and the resulting risk of multicollinearity.

Multicollinearity was evaluated using variance inflation factors (VIFs). Model discrimination was assessed using the area under the receiver operating characteristic curve (AUC), explanatory performance was summarized using Nagelkerke R^2^, calibration was assessed using the Hosmer–Lemeshow test, and Akaike’s information criterion (AIC) was calculated. Internal validation of the primary adjusted model was performed using bootstrap resampling (500 resamples) to obtain an optimism-corrected AUC and the mean calibration slope, and overall accuracy was summarized with the Brier score. For clarity, discrimination (AUC) describes how well the model separates participants with and without multisite pain, whereas calibration (Hosmer–Lemeshow test, calibration slope) describes the agreement between predicted probabilities and observed outcome frequencies; both properties are reported for the primary model.

The robustness of the findings was further examined in three ways: first, the adjusted model was refitted using alternative outcome definitions (≥2 and ≥4 painful regions); second, the number of painful regions was modeled as a count outcome using negative binomial regression, chosen over Poisson regression because of overdispersion (dispersion parameter ≈ 1.3), with incidence rate ratios (IRRs) reported; third, because educational categories were unevenly distributed between the outcome groups, sensitivity models additionally adjusted for educational level were fitted—a conventional indicator-coded model (middle school as the reference category, with separate high-school and university terms) as the principal specification, and the originally planned binary contrast (high school vs. middle school or university) as a secondary check. All tests were two-sided, and statistical significance was defined as *p* < 0.05.

Statistical analyses were performed using IBM SPSS Statistics version 26.0 (IBM Corp., Armonk, NY, USA); the negative binomial count models and the bootstrap internal-validation procedures were performed in Python 3 using the statsmodels and scikit-learn libraries.

## 3. Results

### 3.1. Participant Characteristics

The study included 100 male motorcycle couriers with a median age of 26.0 years (IQR 23.0–29.3) and a median courier employment duration of 5.0 years (IQR 4.0–9.0). Seventy-five participants (75%) were single, 48 (48%) had completed high school as their highest educational level, 76 (76%) worked 8–10 h per day, and 49 (49%) worked five days per week. Sociodemographic and occupational characteristics of the total sample and of the two NMQ pain-burden groups are presented in Table 1.

### 3.2. Nordic Musculoskeletal Pain Burden

Eighty-one participants (81%) reported pain in at least one anatomical region. Based on the number of painful NMQ anatomical regions, 68 participants (68%) reported pain in 0–2 regions and 32 participants (32%) reported pain in ≥3 regions. The number of painful regions ranged from 0 to 8, with an overall median of 1 region (IQR 1–4). Thus, approximately one-third of the study population met the operational definition of multisite musculoskeletal pain.

**Figure 2:**
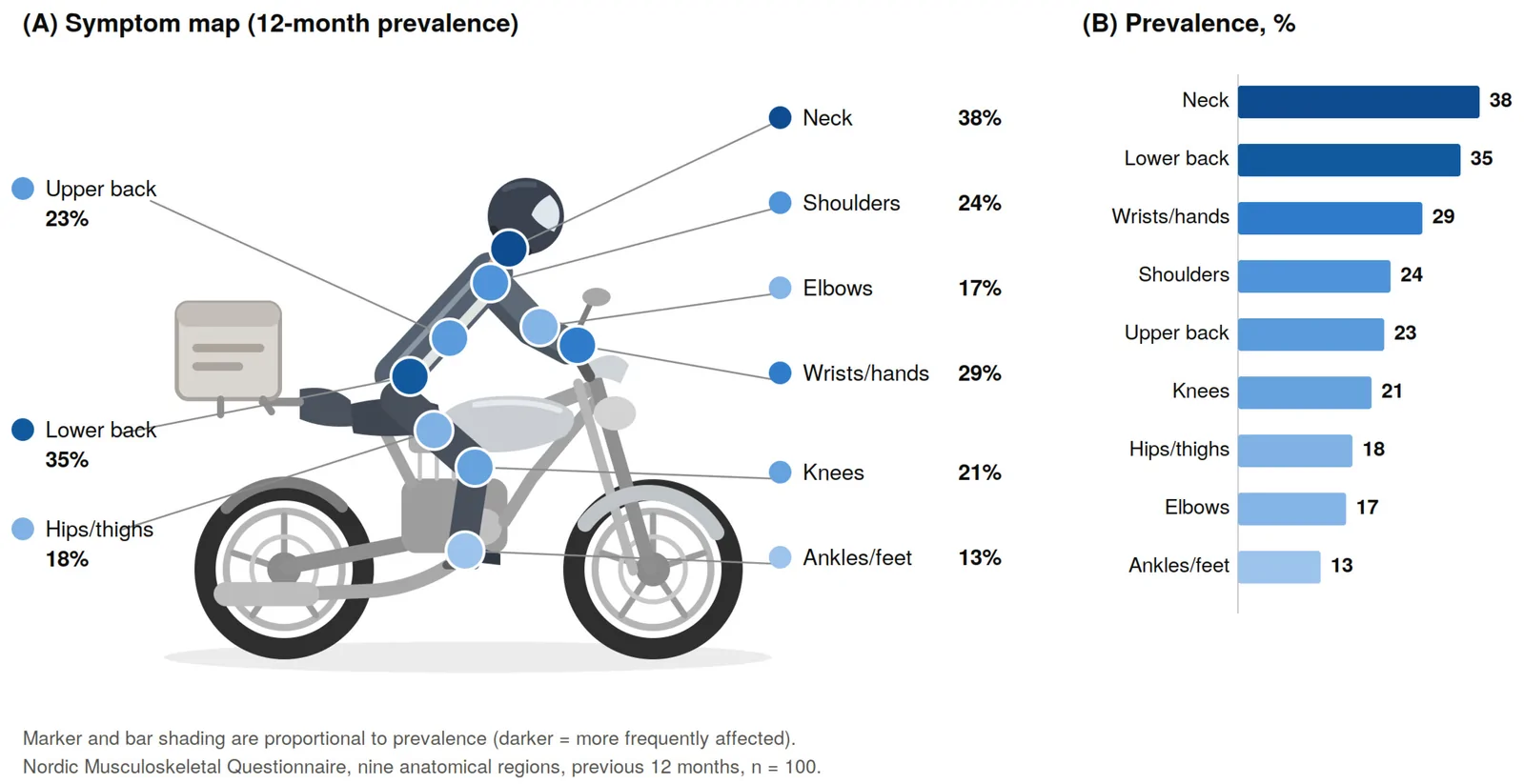
Distribution of musculoskeletal symptoms among motorcycle couriers (n = 100). (**A**) Symptom map showing the nine Nordic Musculoskeletal Questionnaire regions on a rider silhouette; (**B**) 12-month symptom prevalence by anatomical region. Marker and bar shading are proportional to prevalence. The illustration is original artwork created for this study.

The most frequently affected anatomical region was the neck (38%), followed by the lower back (35%), wrists/hands (29%), shoulders (24%), upper back (23%), knees (21%), hips/thighs (18%), elbows (17%), and ankles/feet (13%) (Table 2, Figure 2).

### 3.3. Psychosocial Scale Scores According to Multisite Musculoskeletal Pain

**Figure 3:**
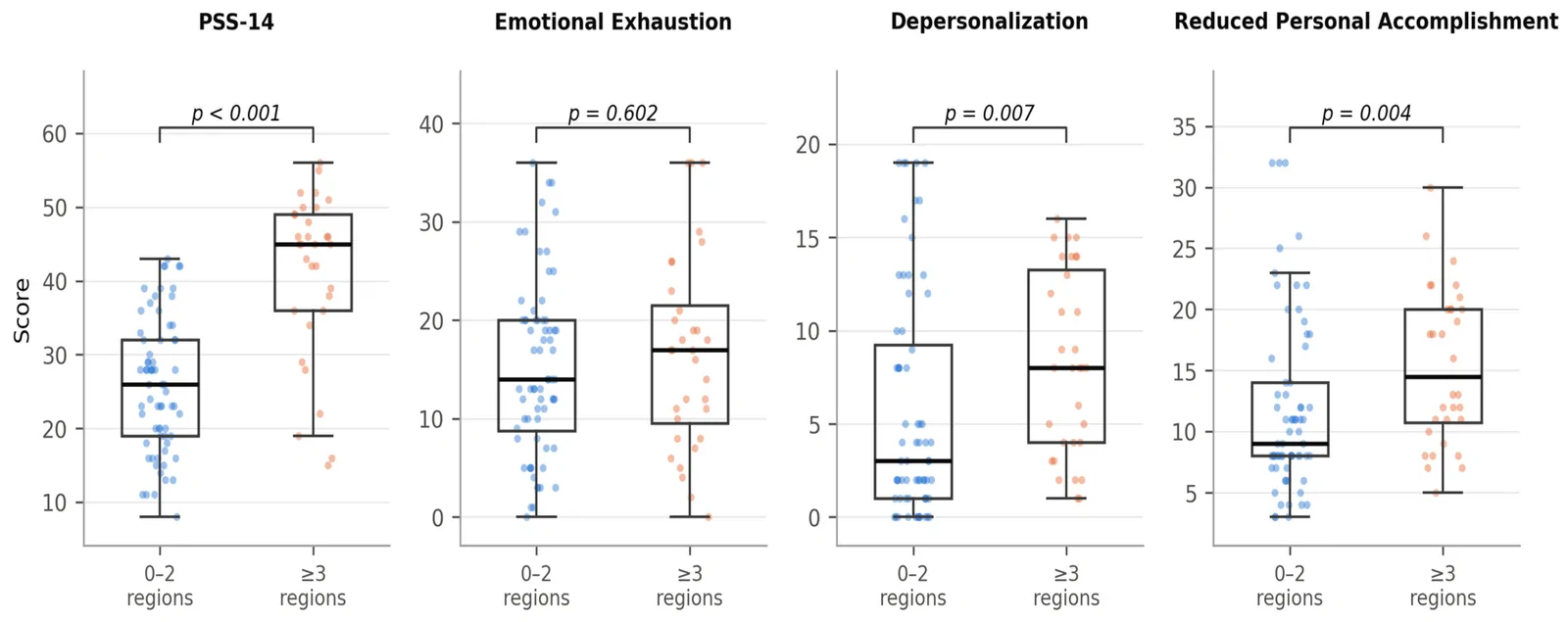
Psychosocial scale scores according to Nordic musculoskeletal pain burden (0–2 vs. ≥3 painful regions). Boxes show medians and interquartile ranges; whiskers extend to 1.5 × IQR; dots represent individual participants. *p*-values from Mann–Whitney U tests.

Comparisons of the psychosocial scale scores between the two pain-burden groups are presented in Table 3 and Figure 3. The most pronounced difference was observed for perceived stress: participants with ≥3 painful regions had markedly higher PSS-14 scores than those with 0–2 painful regions (median 45.0 [36.0–49.0] vs. 26.0 [19.0–32.0]; *p* < 0.001; rank-biserial r = 0.70). Depersonalization (median 8.0 vs. 3.0; *p* = 0.007; r = 0.34) and Reduced Personal Accomplishment (median 14.5 [10.75–20.0] vs. 9.0 [8.0–14.0]; *p* = 0.004; r = 0.36) scores were also significantly higher in the multisite pain group, whereas Emotional Exhaustion did not differ significantly between groups (*p* = 0.602).

### 3.4. Correlations Between Psychosocial Scores and the Number of Painful Regions

Spearman correlations between the psychosocial scale scores and the number of painful anatomical regions are shown in Table 4. The number of painful regions correlated most strongly with PSS-14 (ρ = 0.467, *p* < 0.001), followed by Depersonalization (ρ = 0.315, *p* = 0.001) and Reduced Personal Accomplishment (ρ = 0.274, *p* = 0.006), whereas no significant correlation was observed for Emotional Exhaustion (ρ = 0.154, *p* = 0.125). Emotional Exhaustion and Depersonalization were strongly intercorrelated (ρ = 0.652, *p* < 0.001), and PSS-14 correlated modestly with Depersonalization (ρ = 0.276, *p* = 0.005) and Reduced Personal Accomplishment (ρ = 0.239, *p* = 0.017), supporting the separate analysis of the psychosocial dimensions.

### 3.5. Univariate Logistic Regression

Results of the univariate logistic regression analyses are presented in Table 5. PSS-14 showed the strongest association with multisite musculoskeletal pain: each one-point increase was associated with a 15.5% increase in the odds of pain affecting ≥3 anatomical regions (OR = 1.155, 95% CI 1.091–1.223, *p* < 0.001). Reduced Personal Accomplishment (OR = 1.076, 95% CI 1.012–1.143, *p* = 0.019) and Depersonalization (OR = 1.077, 95% CI 1.002–1.158, *p* = 0.045) were also significantly associated with the outcome in univariate analyses.

No significant associations were identified for age, duration of courier employment, Emotional Exhaustion, daily working hours, or weekly working days.

### 3.6. Adjusted Logistic Regression

**Figure 4:**
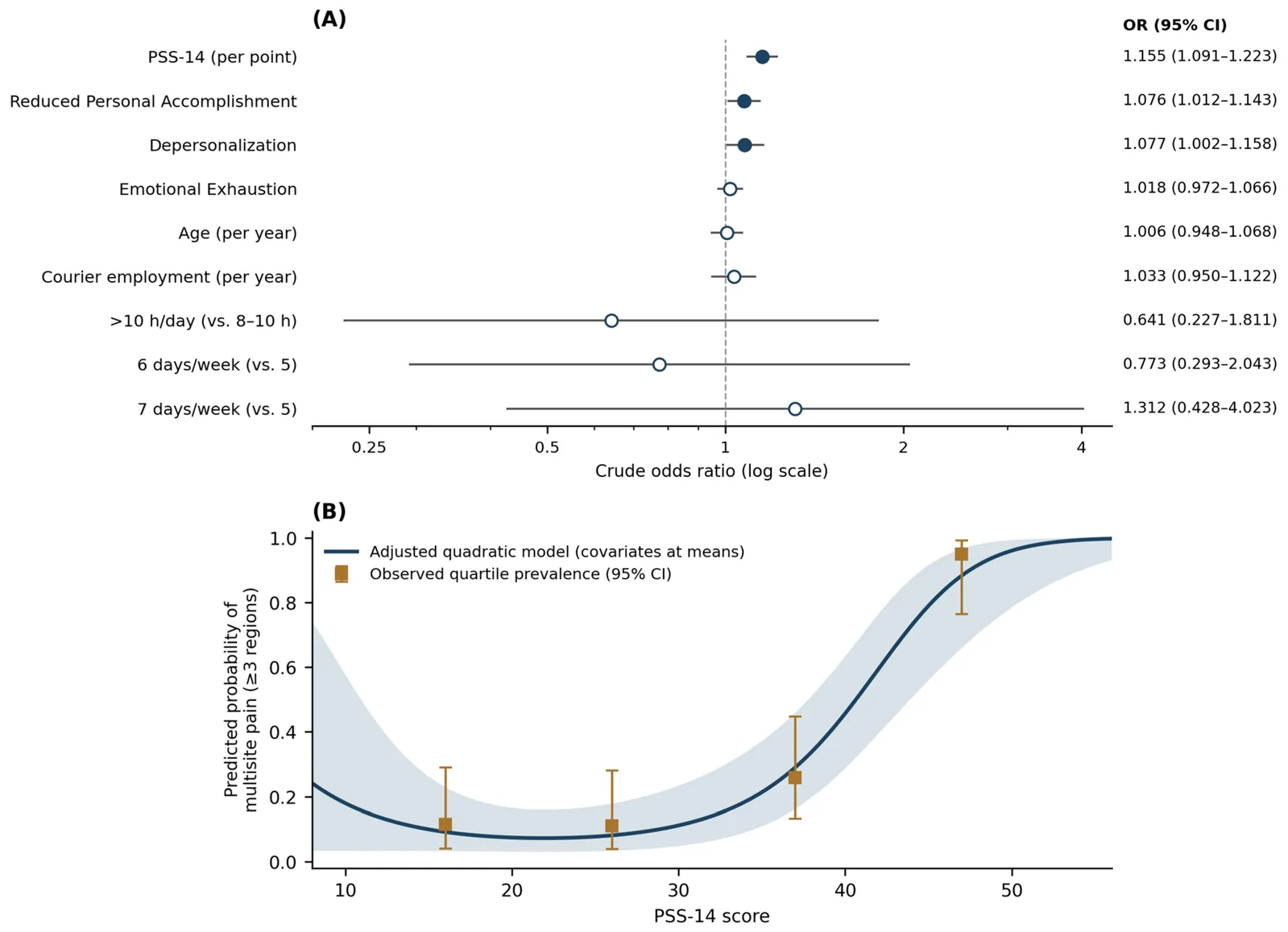
Associations of psychosocial and occupational variables with multisite musculoskeletal pain (≥3 painful regions). (**A**) Univariate logistic regression: crude odds ratios (log scale) with 95% CIs; filled markers denote intervals excluding 1. Hollow markers denote 95% CIs including 1 (not statistically significant).(**B**) Predicted probability of multisite pain across the PSS-14 score from the primary adjusted quadratic model (covariates at their means; shaded area: 95% confidence band); squares show observed quartile prevalences (95% Wilson CIs) at the quartile median score. Uncertainty is wide at the sparse extremes of the score range.

In the adjusted analyses (Table 6, Figure 4), the primary model included linear and quadratic PSS-14 terms together with Reduced Personal Accomplishment and age. Perceived stress was independently associated with multisite musculoskeletal pain in a non-linear fashion: the odds were essentially flat across the lower stress range and rose steeply at high stress (linear term aOR = 1.136 per point at the mean PSS-14 score of 30.7, 95% CI 1.076–1.201, *p* < 0.001; quadratic term aOR = 1.007, 95% CI 1.002–1.012, *p* = 0.005; likelihood-ratio test versus the linear-only specification χ^2^ = 8.30, *p* = 0.004). Equivalently, the model-implied per-point odds ratio increased from approximately 0.97 at a PSS-14 score of 20, to 1.11 at the sample mean, and to 1.36 at the top-quartile boundary (PSS-14 = 43) (Figure 4B); a single per-point odds ratio therefore does not apply uniformly across the score range. Reduced Personal Accomplishment (aOR = 1.059, 95% CI 0.982–1.143, *p* = 0.139) and age (aOR = 1.029, 95% CI 0.952–1.112, *p* = 0.473) were not independently associated with the outcome. For comparison, the linear-only specification—the primary model of the original submission—yielded an average effect of aOR = 1.152 per point (95% CI 1.087–1.220, *p* < 0.001). In an additional model that also included Depersonalization, perceived stress remained the only independent correlate (Depersonalization *p* = 0.599).

The primary (quadratic) model demonstrated strong discrimination (apparent AUC = 0.876), with a Nagelkerke R^2^ of 0.559 and an AIC of 84.39, compared with 0.868, 0.486, and 90.69 for the linear-only model. In contrast to the linear specification, whose significant Hosmer–Lemeshow test (*p* = 0.004) reflected the unmodelled curvature of the stress–pain association, the quadratic model was well calibrated (Hosmer–Lemeshow χ^2^ = 8.06, *p* = 0.43). In bootstrap internal validation (500 resamples), the optimism-corrected AUC was 0.851, the mean calibration slope was 0.86, and the Brier score was 0.114, indicating good discrimination with mild overfitting, as expected with 32 outcome events. VIF values ranged from 1.01 to 1.07, indicating no relevant multicollinearity between the centred linear and quadratic terms or the covariates.

### 3.7. Sensitivity Analyses

The association of perceived stress was robust to alternative outcome definitions (linear PSS-14 specification, retained for comparability across thresholds). When the multisite pain threshold was redefined as ≥4 painful regions (n = 26), the adjusted association of PSS-14 strengthened (aOR = 1.247, 95% CI 1.136–1.369, *p* < 0.001; AUC = 0.936), and it persisted with the lower ≥ 2-region threshold (n = 40; aOR = 1.089, 95% CI 1.044–1.136, *p* < 0.001; AUC = 0.761). Reduced Personal Accomplishment was not independently associated with the outcome at either alternative threshold.

In the education-adjusted sensitivity analyses, perceived stress remained independently associated with multisite pain regardless of how educational level was coded. In the conventional indicator-coded model (middle school as the reference category), the PSS-14 association was essentially unchanged (aOR = 1.136, 95% CI 1.070–1.206, *p* < 0.001); high-school education was independently associated with the outcome (aOR = 6.43, 95% CI 1.70–24.28, *p* = 0.006), whereas university education was not (aOR = 2.53, 95% CI 0.32–19.89, *p* = 0.377; model AUC = 0.887; Nagelkerke R^2^ = 0.540). The originally planned binary contrast (high school vs. middle school or university) led to the same conclusions (PSS-14 aOR = 1.132, 95% CI 1.067–1.202, *p* < 0.001; high school aOR = 5.27, 95% CI 1.58–17.60, *p* = 0.007; AUC = 0.892; Nagelkerke R^2^ = 0.555). Thus, the stress–pain association was not explained by educational differences. The wide confidence intervals of the educational estimates—particularly for the small university category (n = 11)—warrant cautious interpretation, and the likely meaning of this association is considered in the Discussion.

The dose–response pattern underlying the primary non-linear model is detailed in Table 7. The prevalence of multisite pain was 11.5% (3/26) and 11.1% (3/27) in the two below-median PSS-14 quartiles, 25.9% (7/27) in the third quartile, and 95.0% (19/20) in the highest quartile (score range 43–56); the test for linear trend was significant (*p* < 0.001), but the increase was clearly concentrated at the top of the distribution. Relative to the combined below-median reference, the crude odds ratio was 2.74 (95% CI 0.82–9.19) for the third quartile and 148.8 (95% CI 16.8–1320.7) for the highest quartile, and the adjusted categorical model (quartile indicators with Reduced Personal Accomplishment and age) showed the same pattern (third quartile aOR = 2.37, 95% CI 0.69–8.20; highest quartile aOR = 138.99, 95% CI 15.44–1251.15; AUC = 0.874). These quartile-specific estimates derive from sparse cells (only one non-case among the 20 participants in the highest quartile) and are therefore numerically unstable and subject to sparse-data bias: their magnitude should not be taken at face value, and they are presented to convey the ordering and shape of the dose–response pattern rather than precise effect sizes. The excess risk was therefore concentrated in the highest stress quartile, consistent with the threshold-like relationship captured by the quadratic term in the primary model.

In the negative binomial model of the painful-region count including all four psychosocial scores, PSS-14 was the only variable associated with the expected number of painful regions (IRR = 1.041, 95% CI 1.018–1.064, *p* < 0.001), whereas Reduced Personal Accomplishment (*p* = 0.141), Emotional Exhaustion (*p* = 0.435), and Depersonalization (*p* = 0.855) were not. When modeled alone, each one-point increase in PSS-14 was associated with a 4.6% increase in the expected number of painful regions (IRR = 1.046, 95% CI 1.025–1.068, *p* < 0.001). Taken together, these analyses indicate that the association of perceived stress with musculoskeletal pain burden was consistent across all outcome definitions and modeling approaches, whereas the univariate associations of the burnout dimensions did not persist after adjustment.

## 4. Discussion

The present study investigated the musculoskeletal pain burden of motorcycle couriers and examined whether occupational, perceived stress, and burnout-related variables were associated with pain involving multiple anatomical regions, within the JD-R-informed biopsychosocial framework outlined in Figure 1—a framework that guided variable selection and interpretation, but whose pathways were not formally tested here and which includes important occupational exposures that were not measured. Three principal findings emerged. First, multisite musculoskeletal pain was common, with approximately one-third of participants reporting symptoms in three or more anatomical regions. Second, the neck and lower back were the most frequently affected regions. Third, perceived stress was strongly and independently associated with multisite musculoskeletal pain in a non-linear, threshold-like pattern—the excess odds were concentrated at high stress levels rather than increasing uniformly across the score range—and this association was robust across alternative outcome definitions and count-based modeling; Reduced Personal Accomplishment and Depersonalization were associated with the outcome in unadjusted analyses, but these associations attenuated after adjustment for perceived stress, and Emotional Exhaustion showed no detectable association.

The predominance of neck and lower-back symptoms is broadly consistent with the recent delivery-rider literature. In pooled meta-analytic estimates for gig-based food-delivery workers, the lower back (43%) and neck (30%) were among the most affected regions [5], and our region-specific prevalences (lower back 35%, neck 38%) fall within the ranges reported in Thailand [4], Malaysia [6], Shanghai [7], India [8], and Milan [9]. The comparatively prominent neck involvement in our sample may reflect the sustained cervical loading imposed by helmet use and continuous head-up riding postures, in line with evidence that motorbike drivers demonstrate greater neck pain and restricted cervical mobility than car drivers [12] and with the identification of the neck and lower back as vulnerable regions in motorcyclists more broadly [11]. These exposures are biomechanically plausible in motorcycle couriers because prolonged sitting can impose sustained loading on the trunk [13], continuous handlebar control may increase cumulative loading of the cervical and upper-extremity musculature [16], and whole-body vibration is an established correlate of low-back pain in drivers and riders [14,15], having been associated with an approximately 2.7-fold increase in the odds of musculoskeletal disorders among motorcycle delivery riders when exceeding regulatory action values [6].

An important feature of the current investigation was the use of multisite musculoskeletal pain, rather than restricting the outcome to a single anatomical site. Multisite pain shows risk-factor patterns—particularly psychosocial ones—that differ from those of single-site pain [20], and it predicts unfavorable outcomes such as sickness absence and disability more strongly than localized symptoms [22,23]. Approximately one-third of our participants met the ≥3-region definition; although direct comparison with the 46.5% multisite prevalence reported in a general-population survey is limited by differing operational definitions, the burden observed here is clearly substantial for a young, all-male workforce [21]. The recent study by Khera et al. is particularly relevant in this respect: among occupational two-wheeler delivery drivers, psychological distress was significantly associated with the number of body parts affected by musculoskeletal disorders [10]. Our findings closely parallel this observation: perceived stress was associated with multisite pain as a large group difference (median PSS-14 45.0 vs. 26.0), as a monotonic correlation with the number of painful regions (ρ = 0.47), and as an independent association in adjusted models.

The strong association between perceived stress and multisite musculoskeletal pain is consistent with a substantial body of evidence. Prospective studies implicate perceived stress and life stressors in the development of chronic musculoskeletal pain [33], psychosocial stress and musculoskeletal pain co-occur in physically demanding occupations [34], and longitudinal occupational studies have shown that mental stress and hurry predict the persistence of multiple-site pain [24] and that psychosocial work factors influence the number of painful body sites [26]. In our sample, the association was clearly non-linear: the adjusted odds of multisite pain were essentially flat across the two below-median stress quartiles, rose modestly in the third quartile, and increased steeply in the highest quartile (PSS-14 ≥ 43), in which 19 of 20 couriers reported multisite pain. A single per-point odds ratio therefore does not adequately summarize the relationship—which is why the primary analysis models this curvature explicitly—and the association also strengthened when the outcome was redefined as ≥4 painful regions. The apparent strength of the association in the highest quartile must nevertheless be interpreted with particular caution: it rests on 20 participants and a single non-case, so the corresponding estimates are inflated by sparse-data bias and carry very wide confidence intervals, and with 32 outcome events overall the precision of all adjusted estimates is limited. The shape of the relationship—flat at low-to-moderate stress, rising at high stress—is the replicable claim of this study; the specific magnitude at the extreme, and any candidate threshold value, require confirmation in larger samples. It should also be kept in mind that the association links stress appraisals referring to the last month with symptoms recalled over the previous 12 months, a temporal asymmetry that further cautions against mechanistic over-interpretation. Substantively, the threshold-like pattern suggests that stress-related risk becomes pronounced mainly once perceived stress is high, which is also where preventive attention may yield the greatest benefit. Several mechanisms may underlie this relationship: sustained stress may increase muscle tension and sympathetic arousal, impair sleep and physiological recovery, lower pain thresholds through central sensitization-like processes, and reduce engagement in protective behaviors. In platform delivery work specifically, psychological strain arises from occupationally salient mechanisms—algorithmic monitoring, ranking and penalty systems, time pressure, and labor intensity [2,35,36,37]—which may amplify both stress appraisals and biomechanical exposure. Conversely, widespread pain itself is a plausible source of stress, because it interferes with riding comfort, endurance, income-generating capacity, and recovery; a bidirectional relationship is therefore likely (Figure 1, reverse pathway), and longitudinal data indicate that pain and the psychosocial work environment influence each other over time [24].

The burnout dimensions showed a more nuanced pattern. Reduced Personal Accomplishment and Depersonalization were higher in the multisite pain group, correlated with the number of painful regions, and were associated with the outcome in univariate regression, but none of these associations persisted after adjustment for perceived stress. This attenuation describes statistical overlap, not a verified causal pathway: formal mediation was not evaluated, and the cross-sectional design could not support it, so we explicitly refrain from concluding that the burnout dimensions “operate through” perceived stress. Several non-exclusive explanations are plausible. First, the constructs differ in breadth: the PSS-14 captures global appraisals of unpredictability, uncontrollability, and overload across all life domains, whereas the MBI dimensions index work-specific syndromal states; the broader construct may simply lie closer to the common pathways—muscle tension, impaired recovery, central sensitization—through which strain is expressed somatically. Second, the psychosocial processes overlap empirically (PSS-14 correlated modestly with both Depersonalization and Reduced Personal Accomplishment in our sample), so shared variance is partitioned to the stronger correlate in adjusted models. Third, with 32 outcome events, statistical power to detect the smaller independent effects of the burnout dimensions was limited. Associations between burnout and musculoskeletal complaints have been reported across diverse occupational settings: burnout mediated the relationship between job strain and musculoskeletal disorders among hospital nurses [42], and musculoskeletal disorders clustered with emotional exhaustion among office employees [43]. The MBI conceptualizes burnout as a multidimensional construct whose dimensions should be examined separately [30,31], and the personal-accomplishment dimension in particular shows distinct correlates such as occupational self-efficacy [32]; findings for this dimension in relation to musculoskeletal symptoms are heterogeneous across studies [44]. Accordingly, the non-significant adjusted estimates for the burnout dimensions—and for Emotional Exhaustion in particular—should not be read as evidence that these constructs are irrelevant to couriers’ occupational health; they remain plausible components of the strain pathway in Figure 1 that this study was not powered to isolate, and Emotional Exhaustion showed no detectable association with the pain outcome in any analysis, underscoring that the three dimensions behave differently and should not be combined into a single score.

Two further observations deserve explicit comment. First, the magnitude of the stress–pain association observed here (rank-biserial r = 0.70; adjusted AUC ≥ 0.85) is larger than the small-to-moderate effects typically reported in occupational psychosocial research. Because both the exposure and the outcome were self-reported in the same session, common-method variance may have inflated the association, and negative affectivity could have influenced both stress appraisals and symptom reporting; the estimate should therefore be regarded as an upper bound pending replication with independent or objective measures. Second, in the education-adjusted sensitivity analyses, high-school education remained independently associated with multisite pain alongside perceived stress. The conventional indicator-coded model showed that this association was driven by the high-school category relative to middle school, with an imprecise, non-significant estimate for the small university group; such a non-gradient pattern argues against a simple socioeconomic gradient and supports interpreting educational attainment as a proxy for unmeasured differences in platform type, employment arrangement, income security, economic insecurity, workload, access to workplace protections, and reporting behavior. Residual socioeconomic and occupational confounding of the stress–pain association therefore cannot be excluded, particularly given the limited sample size, and this hypothesis-generating association requires confirmation in larger samples.

Interestingly, neither age nor duration of courier employment was associated with multisite pain. This may partly reflect the relatively young age distribution of the sample (median 26 years). Additionally, occupational exposures may be determined more strongly by current daily riding intensity, ergonomic characteristics, load carriage, rest opportunities, and road conditions than simply by chronological age or years employed [4,6]. Consistent with this interpretation, labor intensity and weekly working hours—rather than tenure—were the principal determinants of adverse safety and health outcomes among instant-delivery riders in China [37], and cumulative fatigue and overwork are highly prevalent among riders working long weekly hours [3]. More generally, the absence of statistical significance for age, tenure, Emotional Exhaustion, and the working-time variables should not be interpreted as absence of occupational-health relevance: with 32 outcome events the study had limited power to detect modest associations, and these factors remain theoretically motivated candidates (Figure 1) for larger prospective studies.

In terms of model performance, the distinction between discrimination and calibration is informative. The primary quadratic model discriminated well between couriers with and without multisite pain (apparent AUC = 0.876; optimism-corrected AUC = 0.851) and—unlike the linear specification, whose significant Hosmer–Lemeshow test reflected the unmodelled curvature of the stress–pain association—was well calibrated (Hosmer–Lemeshow *p* = 0.43), meaning that its predicted probabilities agreed with the observed outcome frequencies across the risk range. Nevertheless, the model should be viewed as an association model rather than a clinical prediction tool: it explains a substantial proportion of outcome variance (Nagelkerke R^2^ = 0.559), but external validation would be required before any individual-level use, and unmeasured biomechanical, organizational, and individual factors are likely to contribute to the remaining variation in the outcome.

From an occupational-health perspective, the findings support a multidimensional approach to motorcycle couriers. Interventions directed only toward a single painful body region may overlook workers experiencing widespread or multisite complaints. Meta-analytic evidence indicates that ergonomic interventions can reduce work-related musculoskeletal pain at several sites, including the lower back and neck [45], and such measures—addressing posture, vibration, upper-extremity loading, riding duration, and recovery breaks—may need to be combined with strategies that reduce psychosocial strain, given the strong association between perceived stress and multisite pain observed here. Because the unmeasured exposures in Figure 1 span distinct levels, future work should distinguish biomechanical factors (riding posture, whole-body vibration, load carriage, motorcycle ergonomics), organizational and platform-mediated factors (delivery quotas, algorithmic ranking and penalty systems, scheduling, and rest-break policies), and individual factors (sleep, physical activity, and socioeconomic conditions), since each implies a different intervention lever. At the organizational and policy level, the present findings—together with evidence that platform design features contribute to strain, burnout, and risky riding [35,36], and that fatigue and stress-related factors predict occupational crashes in delivery riders [38]—support concrete measures: reasonable delivery-time expectations and workload caps, protected rest periods within and between shifts, occupational safety education adapted to courier work, accessible injury-reporting mechanisms free of penalty, access to occupational healthcare, and clearer platform and employer responsibility for safe working conditions and couriers’ occupational health rights [1,2,9]. These organizational and policy measures warrant evaluation as research and policy priorities alongside worker-level ergonomic and stress-management interventions.

### Limitations

The present study has several strengths. Musculoskeletal symptoms were evaluated across nine anatomical regions using the standardized Nordic framework rather than focusing exclusively on low-back or neck pain. The use of a multisite pain outcome allowed the cumulative spatial burden of symptoms to be evaluated. Perceived stress and the three MBI dimensions were considered separately, avoiding inappropriate use of a single combined burnout score. The analyses were guided by an explicit conceptual framework (Figure 1)—used heuristically, since its pathways were not formally tested—and the non-linearity of the principal association was modelled explicitly in the primary analysis rather than assumed away. Finally, the robustness of the main findings was examined comprehensively, using alternative outcome definitions, a count-based modeling strategy, education-adjusted sensitivity models with conventional indicator coding, and bootstrap internal validation.

Nevertheless, several limitations should be acknowledged. First, the cross-sectional design prevents causal or temporal conclusions; it cannot be determined whether burnout-related inefficacy increases susceptibility to multisite pain or whether persistent multisite pain contributes to reduced occupational accomplishment. Second, both the exposure and outcome measures were self-reported and collected in the same session; the NMQ is a screening instrument that does not establish a specific musculoskeletal diagnosis, only its general 12-month prevalence item was analyzed (without symptom severity, duration, or activity limitation), and common-method variance may have inflated the observed associations. In addition, the temporal reference periods of the instruments differ: the PSS-14 refers to the last month, whereas the NMQ refers to the previous 12 months, so the exposure measure is not aligned with the full outcome window and the associations should be read as linking current stress appraisals with recalled 12-month symptoms. Third, no formal a priori sample-size calculation was performed, the sample size was relatively modest, particularly because only 32 participants met the ≥3-region outcome, which limited the number of covariates that could be entered simultaneously into logistic regression and resulted in relatively wide confidence intervals for some estimates; bootstrap internal validation corrects for optimism but cannot overcome the information limits of a small number of events. Fourth, the threshold of ≥3 painful regions was an operational definition; although the association of perceived stress was consistent across the ≥2- and ≥4-region thresholds and a count-based model, future studies should evaluate alternative and continuous definitions of multisite pain. Fifth, several potentially important covariates were not available—spanning the biomechanical (body mass index, average riding distance, motorcycle type, vibration exposure, posture, load carried), organizational (number of deliveries, break frequency, platform and employment arrangement, organizational safety practices), and individual (smoking, physical activity level, sleep characteristics, socioeconomic indicators, previous musculoskeletal injury) levels of the framework in Figure 1—so residual confounding, including socioeconomic confounding suggested by the educational findings, cannot be excluded. In addition, all participants were male couriers recruited in a single metropolitan area through non-probability (convenience and snowball) sampling, which may limit generalizability to other settings and to female delivery workers, because couriers recruited through professional networks may share working conditions, platforms, or health characteristics; the exclusion of female couriers in particular means the findings cannot be extended to the growing female segment of the delivery workforce. Sixth, item-level questionnaire data were not retained in the analysis dataset, so sample-specific internal consistency coefficients (e.g., Cronbach’s alpha) could not be calculated. Finally, the regression findings should be interpreted primarily in terms of association rather than prediction.

## 5. Conclusions

Multisite musculoskeletal pain was common among motorcycle couriers, affecting approximately one-third of the study population, and the neck, lower back, wrists/hands, shoulders, and upper back were the most frequently affected anatomical regions. Perceived stress was strongly and independently associated with pain involving three or more anatomical regions, in a non-linear, threshold-like pattern: the adjusted odds were essentially flat at low-to-moderate stress levels and rose steeply in the highest stress quartile (PSS-14 ≥ 43), a relationship that was consistent across alternative thresholds and count-based analyses and that a single per-point odds ratio would misrepresent. Reduced Personal Accomplishment and Depersonalization were associated with the pain burden in unadjusted analyses but not after adjustment for perceived stress, and Emotional Exhaustion, age, and duration of courier employment showed no detectable associations. These results suggest that the musculoskeletal health of motorcycle couriers should be considered within a multidimensional occupational-health framework incorporating both biomechanical exposure and psychosocial strain, with high perceived stress as a priority target for future longitudinal research and workplace- and platform-level interventions.

## Figures and Tables

**Table 1:** Sociodemographic and occupational characteristics of the participants according to Nordic musculoskeletal pain burden.

Characteristic	Total (n = 100)	0–2 Regions (n = 68)	≥3 Regions (n = 32)	*p*
Age, years, median [IQR]	26.0 [23.0–29.3]	26.0 [23.0–30.0]	26.5 [23.0–29.0]	0.844 ^1^
Courier employment, years, median [IQR]	5.0 [4.0–9.0]	5.0 [4.0–9.0]	6.0 [4.0–10.0]	0.294 ^1^
Marital status, n (%)				– ^3^
Single	75 (75.0)	50 (73.5)	25 (78.1)	
Married	25 (25.0)	18 (26.5)	7 (21.9)	
Educational level, n (%)				– ^3^
Middle school	41 (41.0)	37 (54.4)	4 (12.5)	
High school	48 (48.0)	22 (32.4)	26 (81.3)	
University	11 (11.0)	9 (13.2)	2 (6.3)	
Daily working hours, n (%)				0.554 ^2^
8–10 h	76 (76.0)	50 (73.5)	26 (81.3)	
>10 h	24 (24.0)	18 (26.5)	6 (18.8)	
Working days per week, n (%)				0.690 ^2^
5 days	49 (49.0)	33 (48.5)	16 (50.0)	
6 days	33 (33.0)	24 (35.3)	9 (28.1)	
7 days	18 (18.0)	11 (16.2)	7 (21.9)	

IQR, interquartile range. ^1^ Mann–Whitney U test. ^2^ Pearson’s chi-square test. ^3^ Marital status was summarized descriptively and not analyzed inferentially. Educational level was not included in the primary model; its role was examined in dedicated sensitivity analyses (Section 2.7 and Section 3.7). Dashes (–) indicate that no inferential test was performed for these descriptive variables.

**Table 2:** Prevalence of musculoskeletal symptoms by Nordic anatomical region (n = 100).

Anatomical Region	n	%
Neck	38	38.0
Lower back	35	35.0
Wrists/hands	29	29.0
Shoulders	24	24.0
Upper back	23	23.0
Knees	21	21.0
Hips/thighs	18	18.0
Elbows	17	17.0
Ankles/feet	13	13.0

Symptoms refer to the previous 12 months.

**Table 3:** Psychosocial scale scores according to Nordic musculoskeletal pain burden.

Variable	Total (n = 100), Median [IQR]	0–2 Regions (n = 68), Median [IQR]	≥3 Regions (n = 32), Median [IQR]	*p* ^1^	r ^2^
PSS-14	29.0 [20.0–42.0]	26.0 [19.0–32.0]	45.0 [36.0–49.0]	<0.001	0.70
Emotional Exhaustion	14.0 [8.8–20.0]	14.0 [8.8–20.0]	17.0 [9.5–21.5]	0.602	0.07
Depersonalization	4.0 [2.0–11.3]	3.0 [1.0–9.3]	8.0 [4.0–13.3]	0.007	0.34
Reduced Personal Accomplishment	11.0 [8.0–18.0]	9.0 [8.0–14.0]	14.5 [10.8–20.0]	0.004	0.36

PSS-14, 14-item Perceived Stress Scale; IQR, interquartile range. ^1^ Mann–Whitney U test. ^2^ Rank-biserial correlation (effect size).

**Table 4:** Spearman correlation coefficients among psychosocial scale scores and the number of painful anatomical regions (n = 100).

Variable	1	2	3	4
1. PSS-14	—			
2. Emotional Exhaustion	0.106	—		
3. Depersonalization	0.276 **	0.652 ***	—	
4. Reduced Personal Accomplishment	0.239 *	−0.187	0.073	—
5. Number of painful regions	0.467 ***	0.154	0.315 **	0.274 **

PSS-14, 14-item Perceived Stress Scale. * *p* < 0.05; ** *p* < 0.01; *** *p* < 0.001 (exploratory analyses, uncorrected for multiple comparisons).

**Table 5:** Univariate logistic regression analyses for multisite musculoskeletal pain (≥3 painful regions).

Variable	OR	95% CI	*p*
Age, years	1.006	0.948–1.068	0.847
Courier employment duration, years	1.033	0.950–1.122	0.449
PSS-14	1.155	1.091–1.223	<0.001
Emotional Exhaustion	1.018	0.972–1.066	0.454
Depersonalization	1.077	1.002–1.158	0.045
Reduced Personal Accomplishment	1.076	1.012–1.143	0.019
>10 h/day (vs. 8–10 h/day)	0.641	0.227–1.811	0.401
6 days/week (vs. 5 days/week)	0.773	0.293–2.043	0.604
7 days/week (vs. 5 days/week)	1.312	0.428–4.023	0.634

OR, odds ratio; CI, confidence interval; PSS-14, 14-item Perceived Stress Scale.

**Table 6:** Adjusted logistic regression model (primary, non-linear specification) for multisite musculoskeletal pain (≥3 painful regions).

Variable	aOR	95% CI	*p*
PSS-14—linear term (per point) ^1^	1.136	1.076–1.201	<0.001
PSS-14—quadratic term ^1^	1.007	1.002–1.012	0.005
Reduced Personal Accomplishment	1.059	0.982–1.143	0.139
Age, years	1.029	0.952–1.112	0.473

aOR, adjusted odds ratio; CI, confidence interval; PSS-14, 14-item Perceived Stress Scale; AUC, area under the receiver operating characteristic curve. ^1^ The PSS-14 score was mean-centred (mean = 30.7) before the quadratic term was computed; the linear term therefore expresses the per-point odds ratio at the mean score. Model fit: AUC = 0.876; Nagelkerke R^2^ = 0.559; Hosmer–Lemeshow *p* = 0.43. Full fit statistics of the primary model and of the linear-only comparison model are given in Section 3.6.

**Table 7:** Prevalence and odds of multisite musculoskeletal pain across PSS-14 quartiles (n = 100).

PSS-14 Quartile	Score Range	n	Multisite Pain, n (%)	95% CI ^1^	OR (95% CI) ^2^
Quartile 1	8–20	26	3 (11.5)	4.0–29.0	reference
Quartile 2	22–29	27	3 (11.1)	3.9–28.1	reference
Quartile 3	30–42	27	7 (25.9)	13.2–44.7	2.74 (0.82–9.19)
Quartile 4	43–56	20	19 (95.0)	76.4–99.1	148.8 (16.8–1320.7)

PSS-14, 14-item Perceived Stress Scale; OR, odds ratio; CI, confidence interval. ^1^ Wilson score 95% confidence interval for the prevalence. ^2^ Crude odds ratios relative to the combined below-median reference (quartiles 1–2); the corresponding adjusted estimates and a caution regarding the instability of these sparse-cell estimates are given in Section 3.7.

## Data Availability

The datasets generated and analyzed during the current study are available from the corresponding author on reasonable request, together with a variable codebook, subject to ethical and institutional data-sharing requirements. Public deposition of the individual-level dataset is not possible because the written informed consent signed by the participants and the institutional ethics approval cover data sharing for editorial verification and scientific collaboration upon reasonable request, but do not cover open public release of participant-level data.

## Abbreviations

The following abbreviations are used in this manuscript:

NMQ	Nordic Musculoskeletal Questionnaire
PSS-14	14-item Perceived Stress Scale
MBI	Maslach Burnout Inventory
IQR	Interquartile range
OR	Odds ratio
aOR	Adjusted odds ratio
CI	Confidence interval
IRR	Incidence rate ratio
AUC	Area under the receiver operating characteristic curve
AIC	Akaike information criterion
VIF	Variance inflation factor
STROBE	Strengthening the Reporting of Observational Studies in Epidemiology
